# Editorial: Polyunsaturated fatty acids and gut microbiota

**DOI:** 10.3389/fnut.2023.1256817

**Published:** 2023-07-28

**Authors:** Emanuele Rinninella, Lara Costantini

**Affiliations:** ^1^UOC di Nutrizione Clinica, Dipartimento di Scienze Mediche e Chirurgiche Endocrino-Metaboliche, Fondazione Policlinico Universitario A. Gemelli IRCCS, Rome, Italy; ^2^Dipartimento di Medicina e Chirurgia Traslazionale, Università Cattolica del Sacro Cuore, Rome, Italy; ^3^Department of Ecological and Biological Sciences (DEB), Tuscia University, Viterbo, Italy

**Keywords:** microbiota, microbiome, polyunsaturated fatty acids, omega-3, omega-6, metabolites, prebiotics, gut

## Introduction

Diet modulates the gut microbiota composition. On the other side, the gut microbiota influences the actions that some nutrients have in the human body. Among all the nutrients, polyunsaturated fatty acids (PUFAs) showed numerous beneficial effects. Anyway, inconsistent results were found in the literature in relation to the different impacts that omega-6 and omega-3 PUFAs can have on gut microbiota, highlighting that the topic is still undecided (1). For this reason, PUFAs have been candidates prebiotics (i.e., substrates that are selectively utilized by host microorganisms conferring a health benefit) by the International Scientific Association for Probiotics and Prebiotics (ISAPP) waiting to collect convincing weight of evidence in the target host (2). Frontiers in Nutrition published five articles within the present Research Topic. Three papers (i.e., two mouse studies and one human trial) analyzed the actions of PUFAs on gut microbiota and in improving metabolic outcomes, especially for type-2 diabetes mellitus (T2DM) and hyperlipidemia. Two studies analyzed the PUFAs' action on gut microbiota toward chronic inflammation, especially in inflammatory bowel diseases (IBD) and type-1 diabetes mellitus (T1DM).

## PUFAs, gut microbiota, and metabolic diseases

Omega-6 PUFAs showed inconsistent results in the literature in comparison to omega-3 PUFAs and in relation to their action in promoting gut eubiosis. The study of Liu et al. provided further evidence by analyzing the action of plant-derived omega-6 and omega-3 PUFAs in a double-blind randomized trial on 51 patients with marginal hyperlipidemia. In this trial, after the intervention period of 12 weeks with 4 g of omega-3 rich oil (41.2%, constituted of alpha-linolenic acid, ALA, and stearidonic acid) and 4 g of corn oil (44.4% omega-6), the omega-3 intervention group showed a significant decrease in total cholesterol levels.

Moreover, in the intervention group, an increase in the relative abundance of *Bacteroidetes* was found and there was a significant decrease in *Firmicutes*, determining a cumulative decreased ratio between *Firmicutes* and *Bacteroidetes*. Although comparison with healthy controls was absent in the present study, the results confirmed a greater action of omega-3 PUFAs in comparison to omega-6.

As already speculated (2), the earliest evidence about the impact of the different types of saturated and unsaturated fatty acids on gut microbiota seems to reflect the actions already seen at a systemic level, with the following efficacy scale from the least healthy to the healthiest: saturated fats (SFAs) < omega-6 PUFAs < monounsaturated fatty acids (MUFAs) < omega-3 PUFAs. However, it must be considered that the dose and the pathological conditions are important determinants, so this efficacy scale is not likely to be universally valid. Anyway, as reported in the literature (2), MUFAs have a positive action on the gut microbiota, and this was also confirmed in the paper by Anavi-Cohen et al. that analyzed the effect of a high-oleic peanut cultivar against a classic-one (with low oleic acid content) in healthy C57BL/6J mice for 18 weeks. The high oleic content, even if it did not determine significant changes in the metabolic outcome, led instead to an enhanced abundance of *Bifidobacterium, Lactobacillus*, and *Coprococcus* genera.

The paper by Chen et al. supported the concept of the fatty acids' efficacy scale analyzing the effect of sciadonic acid (SA) on gut microbiota. SA belongs to omega-6 FA, even if it lacks the Δ-8 double bond of arachidonic acid (AA). For this reason, SA is not converted in AA in mammals, so SA cannot be converted in the inflammatory eicosanoid mediators (3). The results found showed that the 4-week SA supplementation in C57BL/6J mice, in addition to maintaining glucose homeostasis, was able to reduce the *Firmicutes/Bacteroidetes* ratio and increase the levels of short-chain fatty acids (SCFAs), gut microbiota metabolites with anti-inflammatory potential.

## PUFAs, gut microbiota, and chronic inflammation

Contrary to what was reported above, in the study by Shen et al., a beneficial action of omega-6 was reported. In this study, T1DM was induced in rats with streptozotocin, and 100 μg linoleic acid (LA) or ALA was later supplemented for 21 days. The authors found that ALA and LA can restore many of the abnormalities induced by streptozotocin to improve lipid levels and plasma glucose, as well as restore the *Firmicutes/Bacteroidetes* ratio, improving the SCFAs. Although in literature this effect for ALA is quite proven, it is less clear for LA. However, it should be highlighted that the low supplemented ALA and LA levels, together with the low levels of Δ5 and Δ6 desaturases in T1DM subjects, resulted in a non-significant increase in their respective long-chain metabolites (i.e., AA for LA and EPA/DHA for ALA). This could lead to speculations that at low concentrations, LA cannot be transformed into AA and inflammatory eicosanoids; therefore, it may exert a positive effect on gut microbiota. Conversely, at higher concentrations, LA and omega-6 could be detrimental to their inflammatory cascade, and this is not only at the plasma level but also in the intestinal microenvironment, especially in chronic inflammatory and autoimmune diseases.

In support of the above speculation is the study by Wang et al. where C57BL/6 mice were fed two different diets with high- and low-fat contents from plant oils (low, 4.27%; high, 23.60%), with three different LA/ALA ratios (1:1; 5:1; 50:1) for 12 weeks, and with dextran sodium sulfate-induced colitis. The results showed that a higher LA/ALA ratio was the main determinant of disease aggravation, rather than a high amount of fats in the diet. The lower LA/ALA ratio also determined prebiotic effects as the inhibition of an abnormal expansion of *Proteobacteria* (i.e., a frequently associated symptom of gut dysbiosis) and *Escherichia-Shigella* (i.e., a possible etiological factor of IBD).

**Figure 1:**
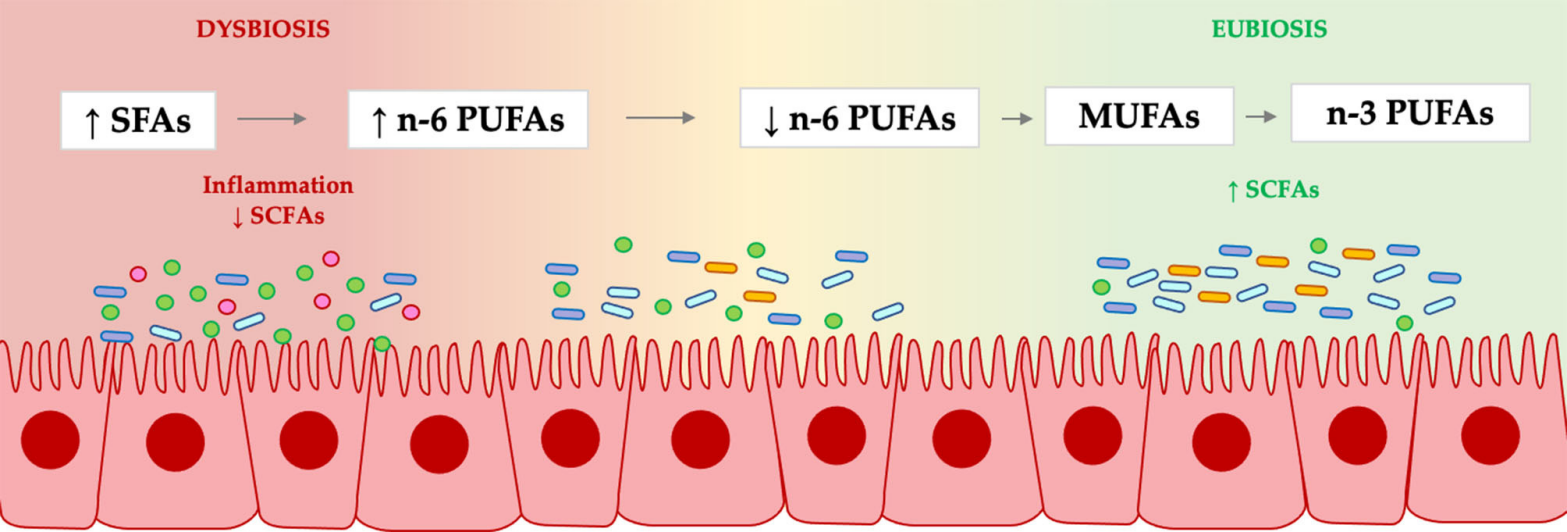
Fatty acid efficacy scale hypothesis.

The aforementioned studies perfectly fit the objectives of this Research Topic, which aimed to highlight the differences between omega-6 and omega-3 prebiotic action and strengthen the fatty acid efficiency scale hypothesis (Figure 1), with a dose effect on gut microbiota.

Further human studies -conducted on large sample sizes and in other inflammatory conditions- are warranted to confirm these exciting results in order to acquire even more information on physiological mechanisms and possible therapeutic implications.

## Author contributions

LC: Conceptualization, Investigation, Project administration, Supervision, Validation, Writing—original draft, Writing—review and editing. ER: Supervision, Validation, Writing—review and editing.
